# Guaiacum in Acute Tonsillitis

**Published:** 1874-12

**Authors:** 


*PHILADELPHIA MEDICAL AND SURGICAL REPORTER—November*, 1874.

GUAIACUM IN ACUTE TONSILLITIS.—In the *Medical and Surgical Reporter*, Dr. Fritzinger recommends the following formula:

| | | |
|---|---|---|
| ℞.—Potass. Chlor., | . . . . . . . | ʒ ij. |
| Spts. Æth. Nitrosi, | . . . . . . . | ℥ ss. |
| Tinct. Guaiaci., | . . . . . . . | ℥ iss. |

M. Ft. Sol. Sig.—A teaspoonful, in sweetened water, every three hours; to be slowly swallowed, to secure its local effects.

